# The Role of Neutrophil-Lymphocyte-Ratio (NLR) and Platelet-Lymphocyte-Ratio (PLR) as a Biomarker for Distinguishing Between Complicated and Uncomplicated Appendicitis

**DOI:** 10.7759/cureus.21446

**Published:** 2022-01-20

**Authors:** Viswa R Rajalingam, Ameer Mustafa, Adewale Ayeni, Fahad Mahmood, Sarah Shammout, Shikha Singhal, Akinfemi Akingboye

**Affiliations:** 1 Colorectal Surgery, Russells Hall Hospital, Dudley, GBR; 2 General Surgery, Russells Hall Hospital, Dudley, GBR; 3 General Surgery, Walsall Manor Hospital, Walsall, GBR; 4 Pathology, Royal Wolverhampton Hospital, Wolverhampton, GBR

**Keywords:** platelet-lymphocyte, neutrophil-lymphocyte, biomarker, complicated appendicitis, uncomplicated appendicitis, acute appendicitis

## Abstract

Introduction

Acute appendicitis (AA) is one of the most common acute general surgical presentations affecting 7% of the population at some point in their lifetime. The ability to assess the risk of complicated appendicitis (CA) from uncomplicated appendicitis (UA) in acute appendicitis (AA) could reduce the associated morbidity and mortality. The value of platelet lymphocyte ratio (PLR) as an inflammatory marker increases when its fluctuations are interpreted along with other complementary hematologic indices, such as neutrophil-to-lymphocyte ratio (NLR), which provides additional information about the disease activity. Hence, we postulated that NLR and/or PLR could serve as a potential surrogate marker in assessing the severity of AA.

Aim

This study aims to investigate the use of PLR and/or NLR as a surrogate biomarker in differentiating uncomplicated from complicated appendicitis.

Material and methods

This retrospective study was conducted at Russells Hall Hospital from January 1, 2017, to December 31, 2020. Data of all patients over age 16 years that had histologically confirmed appendicitis were retrieved. NLR and PLR were calculated from the admission hemogram, and the ratios were compared between uncomplicated (UA) or complicated appendicitis (CA). Cut-off values were calculated using the summarized ROC curve; in addition, the sensitivity and specificity with 95% confidence intervals were determined using SPSS 25.0 (IBM Corp., Armonk, NY).

Results

A total of 799 patients were analyzed, of which 469 (58.7%) were female. The median age was 31.2 years. The difference between NLR and PLR within the two appendicitis groups was significant (P=0.05; Kruskal-Wallis). Cohen’s kappa (degree of inter-rater agreement) between NLR and PLR showed a moderate agreement of 0.589 (P<0.001). We equally demonstrated an exponential relationship between PLR and NLR (R^2 ^=0.510, P<0.05). For UA, the area under the curve (AUC) and the cut-off for NLR and PLR were 0.715, 4.75 with a confidence interval (CI) of 0.678-0.653 and 0.632, 155 with a CI of 0.591-0.672, respectively. For CA, using NLR and PLR, the AUC and cut-off were 0.727, 6.96 with a CI of 0.687-0.768 and 0.653, 180.5 with a CI of 0.602-0.703, respectively; all were significant with a P of <0.001.

Conclusion

NLR and PLR are a reliable, less cumbersome surrogate biomarker for assessing the severity of acute appendicitis.

## Introduction

Acute appendicitis is one of the most common causes of acute abdomen. The lifetime occurrence of this disease is approximately 7%, with a perforation rate of up to 20% [1-2]. Despite the well-known classical symptoms and clinical findings of acute appendicitis, early diagnosis can be sometimes challenging [2]. Diagnosis of acute appendicitis is mainly based upon clinical features with radiological investigations preserved for selected cases and as an ancillary diagnostic tool. Failure to diagnose acute appendicitis at an early stage may result in adverse outcomes, including perforation, which can be associated with significant morbidity and even mortality.

There have been numerous screening and scoring tools to aid the diagnosis of acute appendicitis, including the Alvarado score [3], RIPASA (Raja Isteri Pengiran Anak Saleha Appendicitis) score, and, more recently, the RIFT (Right Iliac Fossa Pain Treatment) score [4-5]. Nevertheless, scoring tools, such as these have been criticised for lack of sensitivity and specificity and not predicting the severity of acute appendicitis [6]. In addition to this, several blood tests are being used to predict appendicitis and its severity. White blood cell (WBC) counts are mostly elevated in patients with appendicitis [7], however, an elevated WBC count has no predictive value in differentiating simple and complicated appendicitis [8-9]. Elevated serum bilirubin has been shown to be a potential marker for perforation of the appendix, but it lacks adequate sensitivity and specificity [10-12]. C-reactive protein (CRP) was found to be superior to bilirubin for anticipation of perforation in acute appendicitis [13]. Identifying a tool or marker that can predict the diagnosis of acute appendicitis and can differentiate between uncomplicated and complicated appendicitis with good sensitivity and specificity is still a subject of interest among many researchers.

Neutrophil-to-lymphocyte ratio (NLR) is a simple inexpensive marker of inflammation, which is easily calculated from the differential WBC counts [14]. NLR provides information regarding two different immune and inflammatory pathways, which may make it a potential marker to predict appendicitis and its severity. A recent meta-analysis demonstrated that NLR predicts both diagnosis and severity of appendicitis [14]. This may have implications for prioritising cases for surgery, for monitoring conservatively treated patients, and for patients who do not routinely undergo CT scans (pregnant or paediatric patients) [14]. We aim to retrospectively evaluate the ability of NLR and/or PLR to differentiate between complicated and uncomplicated appendicitis.

## Materials and methods

Upon gaining approval from our local Clinical Research and Audit department, we conducted a retrospective study in our general surgery department to determine whether PLR /NLR is able to differentiate between uncomplicated or complicated appendicitis in patients with acute appendicitis. Uncomplicated appendicitis is defined as inflammation of the appendix without gangrene or perforation. Complicated appendicitis is defined as gangrenous or perforated appendicitis with or without associated collection.

We screened patients with a radiological or intra-operative diagnosis of acute appendicitis and only analysed patients that eventually had a histological diagnosis of either complicated or uncomplicated appendicitis. All patients aged 16 and over who underwent appendicectomy (laparoscopic/open) between 2017 and 2020 were identified from Hospital Admission Statistics (HAS) data with available postoperative histology. All cases of appendicular neoplasm were excluded.

Outcome 

Our primary outcome is to assess the use of PLR and/or NLR as a biochemical test to differentiate uncomplicated from complicated acute appendicitis.

Data collection 

A comprehensive data collection proforma was used for data collection. The patients were divided into two groups: uncomplicated appendicitis and complicated appendicitis. The data collection proforma included patients’ demographic data, WBC and neutrophil count, lymphocyte and platelet count, how the diagnosis of appendicitis was made, computed tomography (CT) findings if performed, complication rate, length of hospital stay and intra-operative findings where surgical management was considered. For each patient, the data were extracted independently by two team members. Any discrepancies were resolved by discussion between the members. An independent third member was consulted in the event of disagreement.

Data analysis

Analyses were performed using SPSS Statistics for Windows, Version 25.0 (IBM Corp., Armonk, NY). The characteristics of patients were expressed using descriptive statistics. Parameters compatible with normal distribution were described as mean±SD, and non-parametric data are represented as median distribution. For comparisons between the uncomplicated and complicated groups, an independent samples t-test was used for the parameters with normal distribution and the Mann-Whitney U test was used for the parameters with non-normal distribution. A receiver operating characteristic (ROC) curve was utilized to characterize and compare the accuracy of the haematological ratios. The area under the curve (AUC) represented the accuracy of the marker in distinguishing between complicated and uncomplicated AA. Cut-off values were calculated for each biomarker as well as the sensitivity and specificity with 95% confidence intervals and the likelihood ratio were also calculated. P<0.05 is considered to be statistically significant.

Power calculation

In current literature, an NLR of 4.7 has been reported to be a cut-off value for uncomplicated appendicitis and an NLR of 8.8 has been reported to be a cut-off value for complicated appendicitis [14]. With regards to PLR, Pehlivanli and Aydin reported a cut-off of PLR > 140.45 for uncomplicated appendicitis and a PLR > 163.27 for complicated appendicitis [15].

Therefore, to achieve 80% power with a 95% confidence level (CI), it is estimated that a minimum total number of 392 patients will be required. This would require 196 in the uncomplicated appendicitis group and 196 in the complicated appendicitis group.

## Results

A total of 799 patients who were operated on for appendicitis were analysed. The vast majority of patients were operated on laparoscopically, with open appendicectomies accounting for only 2.13% (17/799). These outcomes and the demographics of the patients included are detailed in Table 1.

**Table 1:** Demographic data comparing patients with a normal appendix and uncomplicated and complicated appendicitis (based on histology)

	Normal Appendix (n=255)	Uncomplicated Appendicitis (n=417)	Complicated Appendicitis (n=127)
Mean age in years (SD)	27.22 (10.95)	30.74 (14.35)	40.69 (17.55) (p<0.001)
Mean length of stay in days (SD)	3.44 (2.66)	3.39 (2.69)	5.27 (3.49) (p<0.001)
Number of cases with complications (% of category)	2 (0.78%)	12 (2.88%)	17 (13.39%)
Male	58	201	71
Female	197	216	56
Total	255	417	127

**Figure 1:**
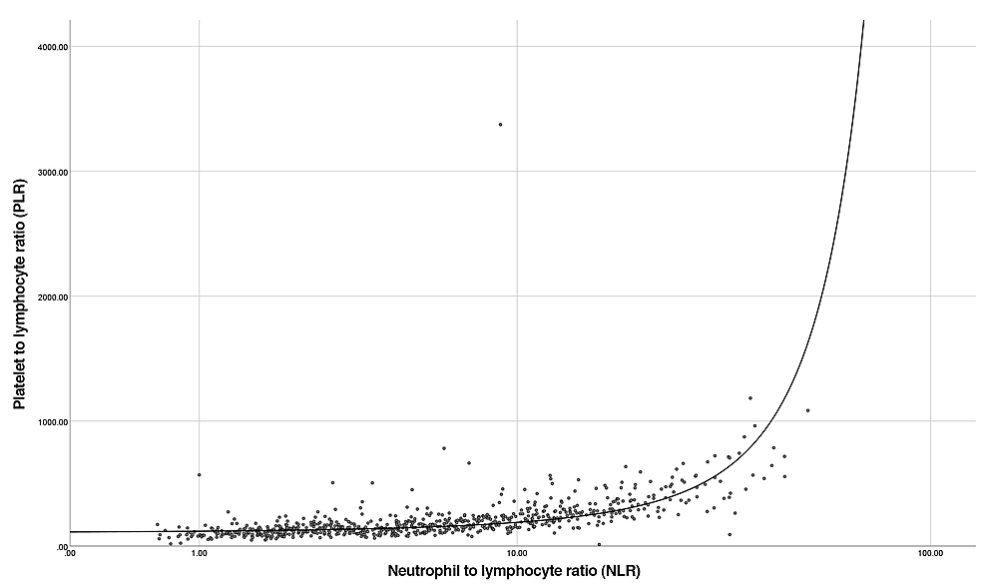
Correlation between NLR and PLR NLR: neutrophil-lymphocyte-ratio; PLR: platelet-lymphocyte-ratio

**Figure 2:**
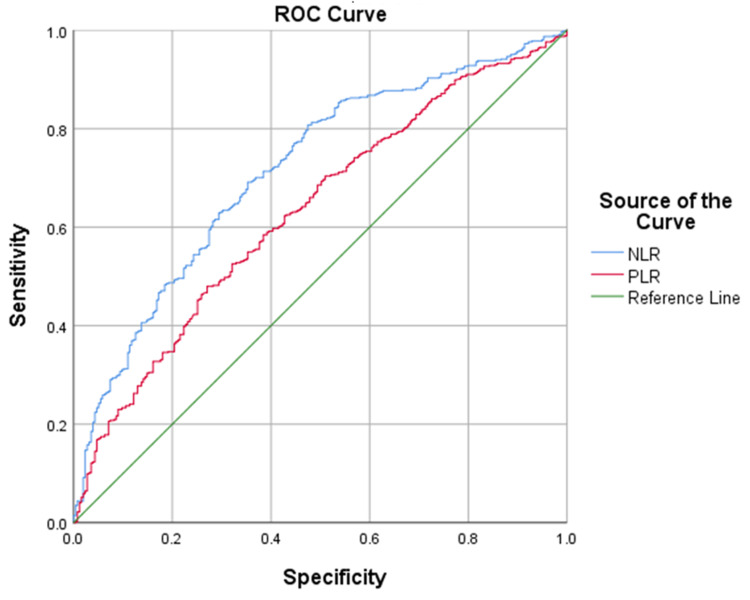
Receiver operating characteristics of NLR and PLR in uncomplicated appendicitis Diagonal segments are produced by ties. NLR: neutrophil-lymphocyte-ratio; PLR: platelet-lymphocyte-ratio

**Figure 3:**
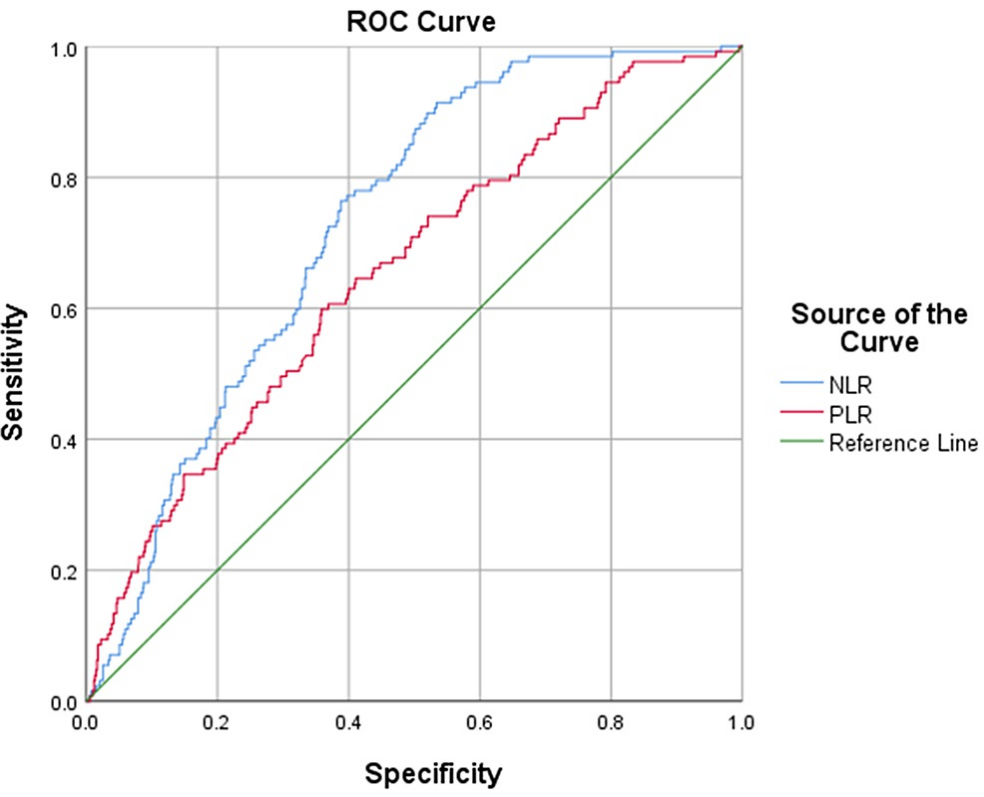
Receiver operating characteristics of NLR and PLR in complicated appendicitis Diagonal segments are produced by ties. NLR: neutrophil-lymphocyte-ratio; PLR: platelet-lymphocyte-ratio

A curve estimation showed that NLR and PLR exhibit an exponential relationship (R2 = 0.510, p<0.001). A Kruskal-Wallis test showed statistical significance between categories for complexity for NLR and PLR (p<0.001). ROC curves for NLR and PLR both had statistically significant AUCs (p<0.001). The correlation between NLR and PLR for uncomplicated and complicated appendicitis is shown in Figure 1. The cut-offs for uncomplicated and complicated appendicitis are detailed in Table 2. These were calculated from the ROC curves of NLR and PLR in complicated and complicated appendicitis (Figure 2 and Figure 3). Cohen’s and Fleiss’ kappa were used to calculate the agreement between NLR and PLR for uncomplicated and complicated appendicitis, respectively. This showed moderate agreement for uncomplicated appendicitis (0.59, p<0.001) and complicated appendicitis (0.543, p<0.001). Crosstabulation for NLR and PLR are shown below in comparison to two commonly used markers of infection (CRP and white cell count). Sensitivity and specificity were also calculated in parallel for NLR and PLR, which were 95.32% and 19.26%, respectively.

**Table 2:** NLR and PLR cut-off values from the ROC curves for uncomplicated and complicated appendicitis NLR: neutrophil-lymphocyte-ratio; PLR: platelet-lymphocyte-ratio

	NLR			
Complexity of appendicitis	Cut-off	AUC	P-value	95% CI
Uncomplicated	4.75	0.715	<0.001	0.678-0.653
Complicated	6.96	0.727	<0.001	0.687-0.768

	PLR			
Complexity of appendicitis	Cut-off	AUC	P-value	95% CI
Uncomplicated	154.98	0.632	<0.001	0.591-0.672
Complicated	180.5	0.653	<0.001	0.602-0.703

## Discussion

Our study has shown that NLR and PLR could be used in differentiating uncomplicated from complicated acute appendicitis with a statistically significant difference. As far as we are aware, we are the first to show concordance between NLR and PLR in discriminating between these diagnoses. Based on our analysis, NLR has a sensitivity of 80.6% and a specificity of 47.2% with a positive predictive value (PPV) of 65.1% and a negative predictive value (NPV) of 66.7% (Table 3). This marker compares favourably to previously described markers such as CRP or bilirubin. McGowan et al. reported that CRP, depending on the cut-off used (>5 to >100), ranges from a sensitivity of 46-94% and a specificity of 32-84%. Its PPV ranges from 16-25% but it has an NPV of 91-97%. In our study, CRP alone has a sensitivity of 74.7% and specificity of 40.7%. Therefore, it can be concluded that CRP is neither sensitive nor specific, but it has a significant negative predictive value in which a normal CRP value is somewhat reassuring.

**Table 3:** Sensitivity and specificity of NLR and PLR (uncomplicated appendicitis) WBC: white blood cell; CRP: c-reactive protein

%	Sensitivity	Specificity	PPV	NPV	Odds ratio	95% CI
NLR	80.6	47.2	65.1	66.7	3.726	2.721-5.103
PLR	75.9	40.8	59.2	60.2	2.176	1.606-2.947
WBC	85.4	39.4	37.7	86.3	3.801	2.557-5.651
CRP	77.1	52.2	78.5	50.2	3.678	2.672-5.063

The NPV of PLR and NLR (66.7% and 60.2%, respectively) lends itself as a useful test to exclude other causes of right iliac fossa pain, thereby avoiding unnecessary laparoscopic appendicectomy, which is not without its complications, albeit minimal.

Based on a recent metanalysis by Hajibandeh et al., NLR > 4.7 is an independent predictor of uncomplicated appendicitis and NLR > 8.8 is an independent predictor of complicated appendicitis [14]. Our cut-off value for NLR in complicated appendicitis of 6.96 is lower than the 8.8 published by Hajibandeh et al. However, we did reach concordance with an NLR of 4.7 in uncomplicated appendicitis. This discrepancy can be potentially explained by the ethnic and regional variation in haemograms as well as the effect of pooled results in meta-analysis [16]. Despite this, however, it is worth noting that our cut-off value of 6.96 remains very highly statistically significant with a p-value of <0.001.

Pehlivanli and Aydin reported that PLR > 140.45 has a sensitivity of 71.4% and specificity of 88.9% to distinguish between appendicitis and a normal appendix, whilst PLR > 163.27 has a sensitivity of 64.3% and a specificity of 67.5% to differentiate complicated appendicitis from uncomplicated appendicitis. This is similar to our data, which suggests PLR > 154.98 has a sensitivity of 75.9% and a specificity of 40.8% for uncomplicated appendicitis and PLR > 180.5 has a sensitivity of 22.4% and specificity of 89% for complicated appendicitis (Table 4). It is worth noting that there are wide variations in the level and unit of measurement of PLR in the literature [17]. Furthermore, heterogeneity between study populations and geographical and ethnic differences can make comparing absolute values difficult [16-17]. In a recent meta-analysis, Liu et al. used a standardized mean difference (SMD) to account for this in the 11 studies included and demonstrated a significant increase in PLR level in uncomplicated appendicitis as compared to controls (SMD: 1.23, 95% CI: 0.88 to 1.59) but was unable to demonstrate this effect when distinguishing between uncomplicated appendicitis and complicated appendicitis (SMD: 2.28, 95% CI: -1.72 to 6.28) [17].

**Table 4:** Sensitivity and specificity of NLR and PLR (complicated appendicitis) NLR: neutrophil-lymphocyte-ratio; PLR: platelet-lymphocyte-ratio

%	Sensitivity	Specificity	PPV	NPV	Odds ratio	95% CI
NLR	26.5	91.6	69.3	63.7	3.958	2.631-5.995
PLR	22.4	89.0	60.6	60.4	2.351	1.594-3.465

However, in our study, we have demonstrated the exponential correlation between PLR and NLR (Figure 1) with a moderate degree of agreement in uncomplicated and complicated appendicitis (Cohen kappa 0.589 and 0.543, p<0.001). This demonstrates that when NLR and PLR are considered together, the reliability of diagnosing both complicated and uncomplicated appendicitis increases significantly.

With the ongoing coronavirus disease 2019 (COVID-19) pandemic, there are greater pressures on our emergency theatres. It is not uncommon for the number of operative procedures to exceed the operational capacity of emergency theatres. NLR and PLR can help risk-stratify patients with acute appendicitis with regards to the timing of surgery to prioritize patients with complicated appendicitis.

It is widely recognized that appendicectomy is an important training operation, and it is usually performed by junior surgeons under supervision [18]. By identifying patients with potentially complicated appendicitis who may not have overt physical signs and parameters prior to surgery, it is possible to identify patients that may have complicated appendicitis and would require operative input from a more senior surgeon. The converse is also true, and NLR and PLR could be used to identify cases that are better suited for training, and this could potentially maximize training opportunities without compromising patient safety. As demonstrated above, NLR and PLR afford us the flexibility of deployment of surgical resources. This allows us to deliver efficient and safe patient care, reflecting the modern standard. The use of CT imaging has seen an increasing role in investigating acute right iliac fossa pain and diagnosing AA [2]. However, in selected populations, such as children and pregnant women, the ionizing radiation risk of CT is difficult to justify. In these patients, NLR and PLR can inform decision-making and plan management.

There is growing interest in conservative management of uncomplicated acute appendicitis in recent years and especially in the recent pandemic. Our findings demonstrate the utility of NLR and PLR in the non-operatively managed uncomplicated acute appendicitis in terms of monitoring the response to conservative management, predicting the risk of complications, and recognizing the failure of conservative treatment.

NLR and PLR are both inexpensive markers of inflammation, which are easily calculated from the differential full blood count (FBC) [17,19]. Hence, it can be presented in standard haemograms, which eliminates user error in manual calculations. This has real-world implications for ease of use and avoids the need to refer to other tools, such as RIFT or Alvarado, which may require reference to online calculators or text-based scoring systems [3,5]. However, it is worth noting as, with all these markers, the timing of the test can significantly alter its sensitivity and specificity. Tests done within hours of the onset of symptoms can be normal only to rise in the hours and days ahead [20]. Given the inherent variability in the timing of presentation to secondary care with respect to their onset of symptoms, it is important to interpret these tests in the context of the presentation. While we recognise this limitation in our study, we believe that a pragmatic approach to the interpretation of results is a vital part of medical practice and further stratifying patients based on their onset of symptoms would limit the real-world applicability of NLR and PLR.

In our study, we have a negative appendicectomy rate of 31.9%. Whilst this is higher than the 26% reported from international centres [21-22], it is in line with rates reported in the UK [23]. It is worth noting that most of the normal appendix removed was in young females who may have concomitant gynaecological pathology contributing to their symptoms. In our practice, we remove a macroscopically normal appendix during diagnostic laparoscopy for RIF pain when no other significant pathology is found. This is a standard and well-recognized practice [2,23]. Our overall complication rate is 3.88% with most of the complications occurring in the complicated acute appendicitis group, with no 90 days mortality. Whilst the morbidity of a negative appendicectomy remains low, it is possible that NLR and PLR could be used in the future to identify these patients prior to surgery.

Our study is based on retrospective data from a single centre and as such, it is not possible to eliminate entirely the possibility of confounding factors such as the timing of the test and machine calibration. Whilst we fell short of 196 patients in the complicated group to meet our power calculation, our results are in line with other publications and thus minimising the potential for a type 2 error. This could be addressed by a multicentre study and would also increase the applicability and potentially highlight differences between different populations groups. Furthermore, we did not assess the combination of NLR and PLR together in our retrospective study due to our sample size. Given our findings, this would be an important question for a future study.

## Conclusions

NLR and PLR ratios are promising markers that can indicate the severity of appendicitis with acceptable sensitivity and specificity, especially when interpreted together. Our data support the use of NLR and PLR to risk-stratify patients with either clinically or radiologically confirmed appendicitis in resource-constrained environments where access to the theatre is limited or where repeat imaging is not immediately available. It can also be used to monitor patients with appendicitis who are being treated conservatively or support the diagnosis in patients where CT imaging is not justified such as children and pregnant women. Further studies are required to assess whether combining NLR and PLR along with other biomarkers, such as CRP, would result in a better predictive value.
